# Generation of Hepatocytes and Nonparenchymal Cell Codifferentiation System from Human-Induced Pluripotent Stem Cells

**DOI:** 10.1155/2022/3222427

**Published:** 2022-11-22

**Authors:** Ying Shi, Jiali Deng, Xiaopu Sang, Yihang Wang, Fei He, Xiaoni Chen, Anlong Xu, Fenfang Wu

**Affiliations:** ^1^State Key Laboratory of Biocontrol, Guangdong Province Key Laboratory of Pharmaceutical Functional Genes, College of Life Sciences, Sun Yat-sen University, Guangzhou, China; ^2^School of Life Sciences, Beijing University of Chinese Medicine, Beijing, China; ^3^Department of Central Laboratory, Shenzhen Hospital, Beijing University of Chinese Medicine, Shenzhen, China

## Abstract

To date, hepatocytes derived from human-induced pluripotent stem cells (hiPSC) provide a potentially unlimited resource for clinical application and drug development. However, most hiPSC-derived hepatocyte-like cells initiated differentiation from highly purified definitive endoderm, which are insufficient to accurately replicate the complex regulation of signals among multiple cells and tissues during liver organogenesis, thereby displaying an immature phenotypic and short survival time *in vitro*. Here, we described a protocol to achieve codifferentiation of endoderm-derived hepatocytes and mesoderm-derived nonparenchymal cells by the inclusion of BMP4 into hepatic differentiation medium, which has a beneficial effect on the hepatocyte maturation and lifespan *in vitro*. Our codifferentiation system suggests the important role of nonparenchymal cells in liver organogenesis. Hopefully, these hepatocytes described here provide a promising approach in the therapy of liver diseases.

## 1. Introduction

Over the past several decades, liver diseases have relentlessly risen to become one of the leading causes of death and illness worldwide [1]. Liver disease is a global health burden, encompassing over 120 million cases of end-stage liver disease and accounting for over 2 million deaths annually worldwide [2]. Liver transplantation is the only curative approach to treat end-stage liver diseases [3]. Currently, cell therapies as alternative treatments to liver transplantation are attracting increasing attention, including hepatocyte transplantation, engineered liver tissues, and bioartificial liver devices [4]. However, the scarcity of donor organs or hepatocytes remains a bottleneck, which restricts the clinical applications.

Human-induced pluripotent stem cells (hiPSCs) have unlimited self-renewal potential, which represent promising cell sources to provide sufficient hepatocytes [5, 6]. However, most of the hiPSC-derived hepatocytes have initiated differentiation from highly purified definitive endoderm (DE) without nonparenchymal cells [7, 8]. It is well known that heterotypic cell-cell interactions between hepatocytes and nonparenchymal cells are essential for the development and maintenance of liver functions [9, 10]. Hepatic stellate cells are the main storage site for vitamin A and are major contributors to liver development [11]. Hepatic endothelial cells maintain liver stiffness by the release of extracellular matrix components [12]. The differentiation system lacking the nonparenchymal cells do not recapitulate the complex cell-cell and cell-matrix interactions in the liver, which limits hepatocyte maturation [13].

To overcome this deficiency, studies mimicked liver development by combining hepatic endoderm cells with endothelial cells and mesenchymal progenitors, resulted in the generation of a liver bud-like structure with improved function [14]. However, these studies which combined allogeneic cells that were isolated from multiple and postnatal individuals still have severe limitations, such as safety concerns, cumbersome operations, and intercellular rejection.

Therefore, there is a need for new approaches to prepare matured hiPSC-derived hepatocytes as a substitute for primary hepatocytes. Here, we reported a protocol that can induce the codifferentiation of endoderm-derived hepatocyte-like cells and mesoderm-derived nonparenchymal cells without any exogenous cells, serum, or genetic manipulation. We mimicked liver development by combining hepatocytes with nonparenchymal cells, which resulted in the generation of a hepatocyte with improved function. This protocol effectively mimics the microenvironment of multiple-cell interactions during liver embryonic development, maybe promoting hepatocyte maturation and longevity.

## 2. Materials and Methods

We followed the methods of Wu et al. [15].

### 2.1. Cell Culture

Human-induced pluripotent stem cell (hiPSC) lines UC and WD were obtained from Guangzhou Institutes of Biomedicine and Health, Chinese Academy of Science. The passage numbers of the cell lines ranged from 41 to 47. Human ESC line H1 was obtained from Wisconsin Cell Research Institute. The cells were cultured on plates coated with Matrigel (BD Biosciences) and maintained in mTeSR™1 medium (STEMCELL Technologies). Before the initiation of hepatocyte-like cell differentiation, hiPSCs were dissociated into clumps using Accutase® solution (Sigma-Aldrich) and were plated onto Matrigel-coated plates and maintained in mTeSR™1 medium. The primary hepatocytes (Lonza) were cultured in HCM.

### 2.2. Culture and Differentiation of the hiPSCs into Monolayer Hepatocyte-Like Cells

When the cells attained a confluence of 40%, mTeSR™1 was replaced with RPMI 1640 (Gibco), containing 2% B27 minus insulin (Gibco), 100 ng/mL activin A (Peprotech), and 50 ng/mL Wnt 3a (R&D System) for 3 days. The medium was then replaced with IMDM (Procell) with 20% knockout serum replacement (Gibco), 1% GlutaMAX™ supplement (Gibco), 1% dimethyl sulfoxide (DMSO) (Sigma-Aldrich), 1% nonessential amino acids (NEAA) (Gibco), and 0.1 mM *β*-mercaptoethanol (*β*-ME) (Sigma-Aldrich) for 4 days. Next, the cultures were placed in IMDM containing 1% GlutaMAX™ supplement, 20 ng/mL oncostatin M (OSM) (Peprotech), 5 ng/mL basic fibroblast growth factor (bFGF) (Peprotech), 0.5 *μ*M dexamethasone (Dex) (Sigma-Aldrich), and 1% insulin-transferrin-selenium (ITS) (Sigma-Aldrich) for 5 days. Finally, the differentiated cells were cultured in hepatocyte culture medium (HCM) (Lonza) supplemented with 10 ng/mL hepatocyte growth factor (HGF) (Peprotech), 0.5 *μ*M Dex, 10 *μ*M lithocholine acid (LCA) (Sigma-Aldrich), 10 *μ*M vitamin K_2_ (Sigma-Aldrich), and 1% ITS for continuous cultivation.

### 2.3. Culture and Differentiation of the hiPSCs into Hepatocyte-Like Cells in Codifferentiation System

When the cell density reaches 100%, mTeSR™1 was replaced with RPMI 1640 containing 2% B27 minus insulin, 100 ng/mL activin A, and 20 ng/mL BMP4 (Peprotech) for 4 days. The medium was then replaced with IMDM with 20% knockout serum replacement, 1% GlutaMAX™ supplement, 1% DMSO, 1% NEAA, and 0.1 mM *β*-ME for 5 days. The following steps are the same as the monolayer hepatocyte-like cells.

### 2.4. Culture and Differentiation of the hiPSCs into Hepatocyte-Like Cells in Optimizing Codifferentiation System

When the cells attained a confluence of 100%, mTeSR™1 was replaced with RPMI 1640 containing 2% B27 minus insulin, 100 ng/mL activin A, and 20 ng/mL BMP4 for 4 days. The medium was then replaced with RPMI 1640 containing 2% B27 (Gibco), 30 ng/mL FGF4 (Peprotech), and 20 ng/mL BMP2 (Peprotech) for 5 days. Next, the cultures were placed in RPMI 1640 containing 2% B27, 20 ng/mL HGF, and 20 ng/mL KGF (Peprotech) and cultured for a further 5 days. Finally, the differentiated cells were cultured in HCM supplemented with 10 ng/mL OSM and 0.1 *μ*M Dex for continuous cultivation.

### 2.5. Alkaline Phosphatase Staining

Staining of hiPSCs for alkaline phosphatase was achieved with the Alkaline Phosphatase Detection Kit (Millipore, SCR004).

### 2.6. Immunofluorescence Assay

Cells were fixed with 4% paraformaldehyde (PFA) (Sangon Biotech) for 20 min at room temperature (RT), blocked and permeabilized with 5% donkey serum (The Jackson Laboratory), and 0.3% triton X-100 (Sigma-Aldrich) in phosphate-buffered saline (PBS) (Solarbio) at RT for 1 h. Cells were washed 3 times with PBS for 5 min at RT between each step. Next, the cells were incubated with primary antibodies in 3% donkey serum and 0.3% triton X-100 at 4°C overnight and secondary antibodies in 1% donkey serum at RT for 1 h. Finally, DAPI (Sigma-Aldrich) was used to stain the cell nuclei. Cells were washed 4 times with PBS for 5 min at RT after primary and secondary antibodies and DAPI staining. All immunofluorescence images were acquired using the Leica Dmi8 inverted microscope. LAS X software (Leica) was used for image processing. The complete list of the primary and secondary antibodies used is provided in supplemental materials (see Supplementary 2).

### 2.7. Flow Cytometry

Samples were incubated with Accutase/Trypsin-EDTA solution (Sigma-Aldrich) at 37°C until the cells began to dissociate. Cells were centrifuged and resuspended in 4% PFA at RT for 15 min and centrifuged and resuspended 4 times with Intracellular Staining Permeabilization Wash Buffer (BioLegend) for 5 min at RT. Cells were then incubated with primary antibodies at 4°C overnight and secondary antibodies at RT for 1 h. The results were analyzed using the FACSCalibur flow cytometer (BD Biosciences) and were processed using FlowJo VX.

### 2.8. RNA Extraction and Quantitative PCR

Total RNAs were isolated from cells by TRIzol™ Reagent (Invitrogen). The cDNAs was synthesized with 1 *μ*g of total RNAs using PrimeScript RT reagent Kit with gDNA Eraser (Takara). Quantitative PCR was performed with TB Green Premix Ex Taq II (Takara) using the LightCycler 480 II (Roche). Relative quantification was performed against a standard curve, and the values were normalized against the housekeeping gene, and glyceraldehyde 3-phosphate dehydrogenase (GAPDH). Primers used are listed in supplemental materials (see Supplementary 2).

### 2.9. Oil Red O and Periodic Acid-Schiff Staining

Oil Red O (ORO) and periodic acid-Schiff (PAS) (all from Solarbio) staining were performed in accordance with the manufacturer's instructions.

### 2.10. Cellular Uptake and Release of Indocyanine Green

Cells were incubated with 1 mg/mL indocyanine green (ICG) (Sigma-Aldrich) for 6 h at 37°C. The medium containing ICG was then discarded, and the cells were washed with PBS. Next, cellular uptake of ICG was examined using the microscopy. Cells were finally returned to the culture medium and incubated for a further 12 h at 37°C to determine the release of cellular ICG.

### 2.11. Senescence *β*-Gal Staining

The cultures were fixed with 4% PFA at RT for 20 min, then *β*-Gal staining (Cell Signal) was performed in accordance with the manufacturer's instructions.

### 2.12. TUNEL Staining

The cells were fixed with 4% PFA at RT for 30 min, then TUNEL staining (Beyotime) was used according to manufacturer's instructions.

### 2.13. AAT and ALB Assay

Human alpha1-antitrypsin (AAT) and albumin (ALB) production were detected using ELISA kits (Elabscience) in accordance with the manufacturer's instructions. Cells were incubated in fresh medium for 24 h, and subsequently, the cell supernatant was collected at different time points and analyzed for albumin secretion. Cells were either trypsinized and counted using a hemocytometer or lysed for protein content assay with a BCA Protein Assay Kit (Beyotime). Luminescence was measured using a luminometer (GloMax Discover, Promega).

## 3. Results

### 3.1. Screening of an Optimal Culture Medium for Maintenance of hiPSC-Derived Hepatocytes

**Figure 1:**
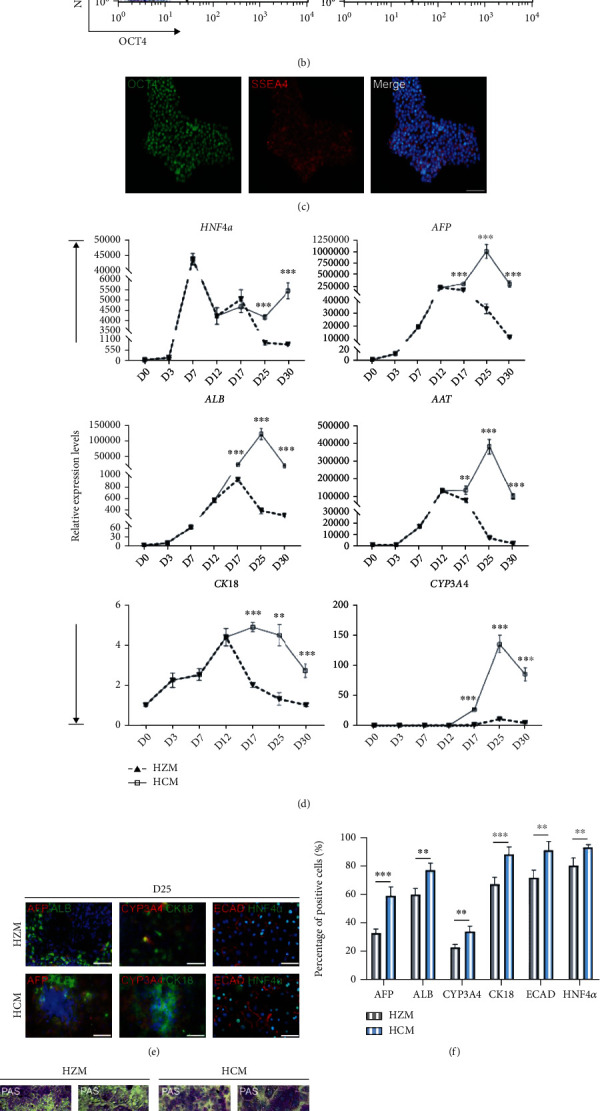
Screening of an optimal culture medium for maintenance of hiPSC-derived hepatocytes. (a) Workflow representation of the differentiation procedure. Sequential morphological changes (day 0-25) of hiPSC differentiation into hepatocytes. Scale bars, 50 *μ*m. (b) Flow cytometry showed the coexpression of NANOG and OCT4 about 97% in day 0 hiPSCs. (c) Immunofluorescence showed the coexpression of SSEA4 and OCT4 in the most of day 0 hiPSCs. Scale bars, 50 *μ*m. (d) Quantitative PCR analysis showed significantly higher expression of mature hepatocyte markers over differentiation in the HCM group when compared with the HZM group.  ^∗^*p* < 0.05,  ^∗∗^*p* < 0.01,  ^∗∗∗^*p* < 0.001, and *n* = 3. (e, f) Immunofluorescence showed the coexpression of mature hepatocyte markers in day 30 cells, respectively. Scale bars, 50 *μ*m.  ^∗^*p* < 0.05,  ^∗∗^*p* < 0.01,  ^∗∗∗^*p* < 0.001, and *n* = 3. (g) Hepatocytes performed periodic acid-Schiff staining (PAS; top), Oil Red O staining (ORO; middle), and indocyanine green uptake/release analysis (ICG; bottom). Scale bars, 25 *μ*m. (h) Bright field images showed the morphological changes of hepatocytes in HCM and HZM (days 35-45). Scale bars, 25 *μ*m. (i) Bright field images of senescence *β*-Gal staining in hepatocytes during days 35-45. Scale bars, 25 *μ*m.

In 2017, Wang et al. reported an elegant protocol for robust and highly efficient generation of hepatocytes derived from hiPSCs using a serum-free-based procedure [16]. This protocol through 3-stage approach to generate hiPSC-derived hepatocytes and sequential morphological changes is outlined in Figure 1(a) and Supplemental Figure 2A.

It is important to select the optimal medium for specific application to generate functionally matured hepatocytes. Recent studies compared five commercial media suggest that hepatocyte culture medium (HCM) is most suitable for culturing primary human hepatocytes [17]. In order to obtain better culture conditions to improve hepatocyte function and prolong survival *in vitro*, we firstly compared HCM to original protocol (Hepato ZYME, HZM) during maturation stage.

The pluripotency of stem cells is an important prerequisite to ensure their directed differentiation. Before differentiation, we examined the pluripotency of hiPSCs at day 0 by flow cytometry and immunofluorescence and found that more than 90% of cells coexpressed pluripotent marker (OCT4, NANOG, or SSEA4) (Figures 1(b) and 1(c)). To verify the accuracy of our results, we used hiPSC-UC, hiPSC-WD, and H1 (data not shown) cell lines for this study (Supplemental Figure 1A-D).

HiPSC-derived hepatoblasts (day 12) were cultured with the HZM or HCM media until day 30 during differentiation. The gene expression levels of hepatocyte markers (*HNF4α*, *AFP*, *CK18*, *AAT*, *CYP3A4*, and *ALB*) in hiPSC-derived hepatocytes were significantly increased by using HCM compared to HZM (Figure 1(d), Supplemental Figure 2B). Immunofluorescence results showed coexpression of sevaral proteins (AFP, ALB, CYP3A4, CYP2D6, ECAD, HNF4*α*, and CK18) in day 25 (Figures 1(e) and 1(f), Supplemental Figure 2C). These results indicated that HCM may be more suitable for maintenance of hiPSC-derived hepatocytes.

Subsequently, we analyzed the hepatocyte function cultured in HCM or HZM media via periodic acid-Schiff (PAS), Oil Red O (ORO), and indocyanine green (ICG) staining in day 25 (Figure 1(g), Supplemental Figure 1E, 2D). In the HCM group, more lipid droplets and glycogen accumulated around the hepatocytes, which indicated a better lipid metabolization and glycogen synthesis ability than those in the HZM group. Meanwhile, the hepatocytes cultured in the HCM medium showed higher ICG uptake and release capacity than the HZM group. ELISA assay showed that the amount of secreted ALB and AAT was more strongly in the HCM group (Supplemental Figure 2E).

To examine the role of HCM in antisenescence, we performed *β*-Gal staining in HBOs. Phase-contrast and staining images showed better morphology and less *β*-Gal-positive cells in the HCM group than those in the HZM group for hepatocytes during day 35 to day 45 (Figures 1(h) and 1(i), Supplemental Figure 1F).

In conclusion, the HCM medium is more suitable for maintaining the function of hepatocytes derived from hiPSCs by comparing with the original differentiation protocol. Therefore, HCM was subsequently chosen as the medium for culturing hepatocytes at the maturation stage.

### 3.2. Codifferentiation of Hepatocytes and Nonparenchymal Cells

**Figure 2:**
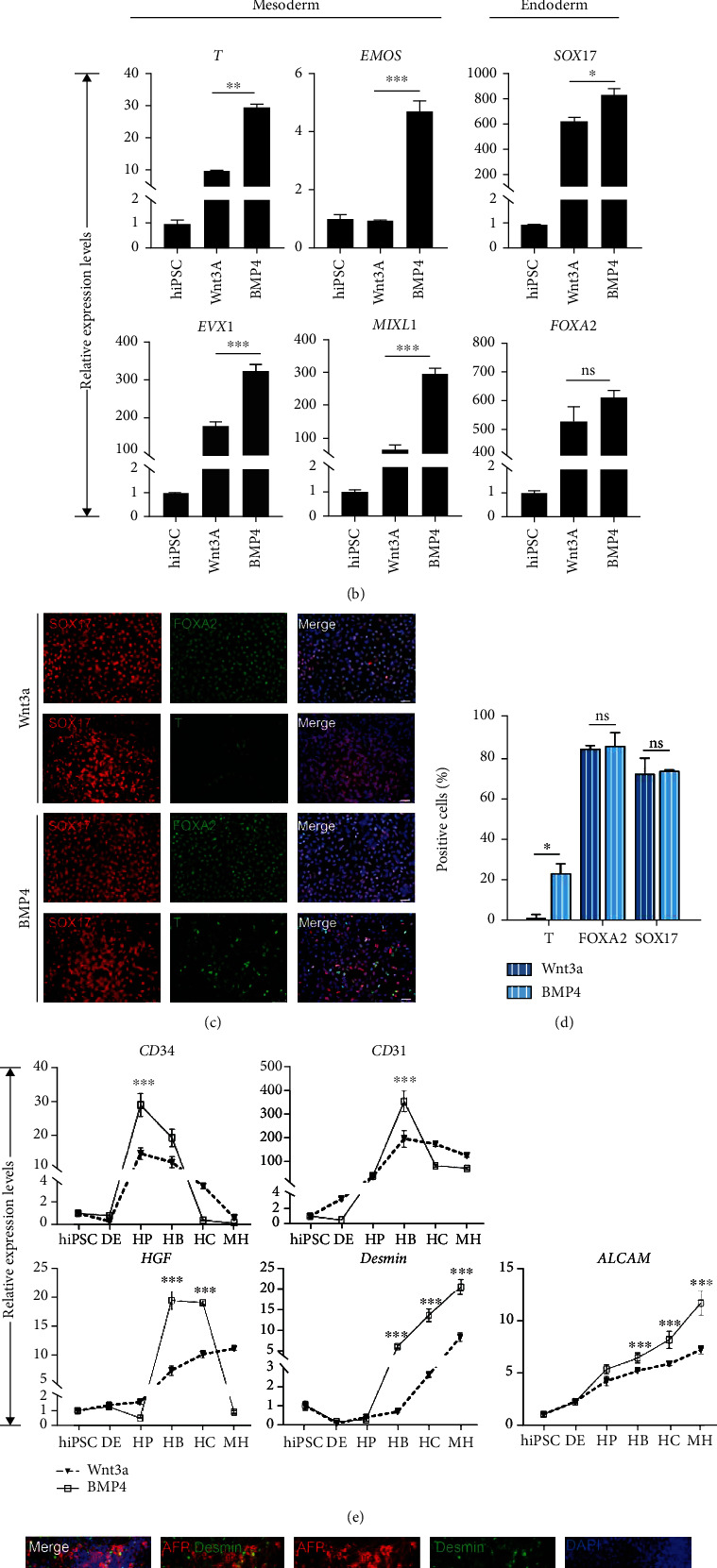
Codifferentiation of hepatocytes and nonparenchymal cells. (a) Schematic representation of the hepatocyte differentiation procedure during day 0 to day 30. The bright field images showed the sequential morphological changes (days 0-30) of hiPSC differentiation into hepatocytes. Scale bars, 50 *μ*m. (b) Quantitative PCR analysis and immunofluorescence (c, d) showed that addition of BMP4 into endoderm medium resulted in main endoderm and a small part mesoderm commitment. Scale bars, 50 *μ*m.  ^∗^*p* < 0.05,  ^∗∗^*p* < 0.01,  ^∗∗∗^*p* < 0.001, and *n* = 3. (e) Quantitative PCR analysis showed endothelial cell marker gene expression levels during hepatocyte differentiation.  ^∗^*p* < 0.05,  ^∗∗^*p* < 0.01,  ^∗∗∗^*p* < 0.001, and *n* = 3. (f) Immunofluorescence image showed endothelial cells in hepatic progenitors and hepatoblast stage in the BMP4 group. Scale bars, 50 *μ*m.

Switching to HCM medium significantly improves hepatocyte function and survival time compared to the original differentiation protocol; however, we still hope to achieve a new breakthrough by simulating the liver development process *in vivo*. The human liver is composed of parenchymal cells (hepatocytes and cholangiocytes) and nonparenchymal cells (endothelial cells, Kupffer cells, hepatic stellate cells and liver resident, and infiltrating lymphocytes), which come from different germ layers [11]. The earliest stage of liver development depends on the careful orchestration of signals between endodermal epithelial, mesenchymal, and endothelial progenitors [18]. We expect to optimize the endoderm medium and thus retain a part of the mesodermal commitment. BMP4 has been applied to the induction of a variety of endothelial cells [19, 20]. We hypothesized that the replaced actin A with BMP4 in endoderm medium would allow for multiple cellular codifferentiation. We modified induction protocol and sequential morphological changes as outlined in Figure 2(a), Supplemental Figure 3A. Subsequently, we utilize q-PCR and immunofluorescence analysis to check whether the mesoderm was retained at the endodermal stage or not. Astonishingly, the cells with BMP4 treatment showed robust upregulation of genes associated with endoderm (*FOXA2* and *SOX17*) and mesoderm (*T*, *EVX1*, *EMOS*, and *MIXL1*) (Figure 2(b)). Immunofluorescence at day 4 showed that most cells expressed DE markers FOXA2 and SOX17, with a small part of cells expressing mesoderm marker T (Figure 2(c)). The number of T positive cells in the BMP4-treated group was significantly more than that in the Wnt3a-treated group (Figure 2(d)). This result indicated that the addition of BMP4 achieves codifferentiation of mesoderm and endoderm.

We also detected whether mesodermal-derived cells are accompanied with hepatocyte differentiation through q-RCR and immunofluorescence analysis. The q-RCR result showed that the expression of endothelial cell markers (*CD34*, *CD31*, *ALCAM*, and *Desmin*) gradually increases, and their expression levels were higher in the BMP4-treated group during hepatocyte differentiation (Figure 2(e)). A similar result was obtained in another cell line, iPSC-WD (Supplemental Figure 3B). Immunofluorescence showed that endothelial cells (Desmin- and *α*SMA-positive cells) were present in BMP4-induced conditions in hepatocyte stage (Figure 2(f), Supplemental Figure 3D).

In conclusion, our results indicated that BMP4 added in endoderm medium could retain a part of mesoderm cells, which could differentiate along with the hepatocytes. However, it is unclear whether these endothelial cells promote hepatocyte maturation.

### 3.3. Hepatocyte Functional Assay after Hepatocyte and Nonparenchymal Cell Codifferentiation

**Figure 3:**
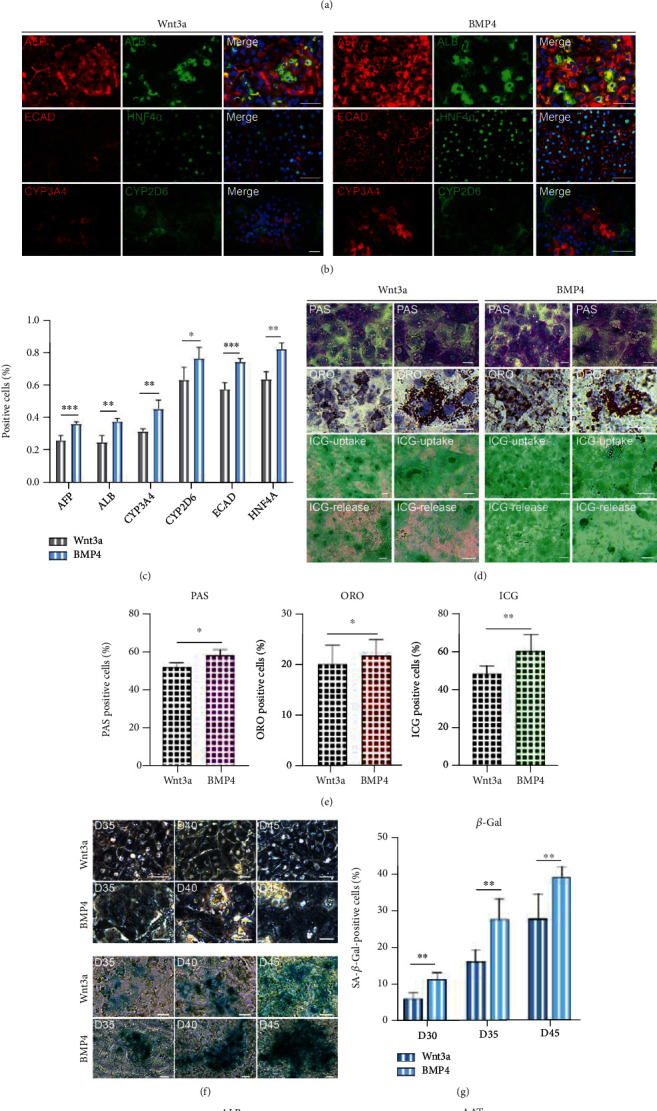
Hepatocyte functional assay after hepatocyte and nonparenchymal cell codifferentiation. (a) Quantitative PCR analysis showed the expression levels of mature hepatocyte markers.  ^∗^*p* < 0.05,  ^∗∗^*p* < 0.01,  ^∗∗∗^*p* < 0.001, and *n* = 3. (b, c) Immunofluorescence showed the coexpression of mature hepatocyte markers in day 25 cells. Scale bars, 50 *μ*m.  ^∗^*p* < 0.05,  ^∗∗^*p* < 0.01,  ^∗∗∗^*p* < 0.001, and *n* = 3. (d, e) Hepatocytes performed PAS staining (top), ORO staining (middle), and ICG uptake/release analysis (bottom). Scale bars, 25 *μ*m.  ^∗^*p* < 0.05,  ^∗∗^*p* < 0.01,  ^∗∗∗^*p* < 0.001, and *n* = 3. (f, g) Phase-contrast images of senescence *β*-Gal staining in hepatic cells during days 35-45 treated with Wnt3a or BMP4. Scale bars, 25 *μ*m.  ^∗^*p* < 0.05,  ^∗∗^*p* < 0.01,  ^∗∗∗^*p* < 0.001, and *n* = 3. (h) Production of ALB and AAT in day 35 hepatocytes. Primary hepatocytes were used as positive controls; hiPSCs were used as negative control.  ^∗^*p* < 0.05,  ^∗∗^*p* < 0.01,  ^∗∗∗^*p* < 0.001, and *n* = 3.

To confirm whether mesoendodermal codifferentiation can promote hepatocyte maturation, we analyzed the mRNA and protein levels of the marker genes during hepatocyte differentiation. The q-PCR results showed that the expression levels of hepatocyte-associated genes significantly increased as the time of differentiation continues. More importantly, the expression levels of *ALB*, *AAT*, *AFP*, *HNF4α*, and *CYP3A4* were significantly increased in the BMP4-treated group compared to the Wnt3a-treated during differentiation (Figure 3(a), Supplemental Figure 3C). The results was also confirmed by immunofluorescence in day 25 (Figures 3(b) and 3(c)). These results indicated that mesoendodermal codifferentiation may promote hepatocyte maturation.

To further validate these results, we performed analysis on hepatocyte function. Both PAS, ORO, and ICG staining showed that BMP4 treatment exhibited better function compared to the Wnt3a-treated group in day 25 (Figures 3(d) and 3(e), Supplemental Figure 3E). This result suggested to us that BMP4 induced of hepatocytes with faster maturation may also cause faster senescence. The bright field images showed that BMP4-treated cells gradually lose their polygonal morphology and have a high nucleoplasmic ratio, which is an important sign of hepatocyte senescence during days 35-45 [21]. Phase-contrast staining images showed less *β*-Gal^+^ cell population in the Wnt3a group than those in the BMP4 group for hepatic cells during differentiation (Figures 3(f) and 3(g)). To further compare the liver function under the two induction conditions, the secretion of ALB and AAT was detected by ELISA (Figure 3(h)). Notably, the ability of hepatocytes induced by BMP4 protocol to secrete ALB and AAT was higher than that of Wnt3a protocol.

In conclusion, the addition of BMP4 to the endoderm medium allows for mesoendodermal codifferentiation. This protocol through multicellular codifferentiation effectively promotes the maturation of hepatocytes. However, BMP4-induced hepatic cells exhibited faster senescence. Therefore, a further effort is still needed to obtain hepatic cells with high maturity and long survival time *in vitro*.

### 3.4. Optimization of the BMP4-Treated Differentiation Protocol Significantly Promotes Nonparenchymal Cell Production

In order to obtain more functional hepatocytes with longer survival time, we further optimized the original BMP4 differentiation protocol. It is well known that liver originates from the endoderm germ layer. Optimization and specification of the earliest differentiation step, which is the definitive endoderm (DE), is very important for generating hepatocytes [22, 23]. We assumed that the maturation of hepatocytes in the BMP4-treated group was inferior to the Wnt3a-treated group, which may be due to the short duration of stage I and stage II. Therefore, we also made adjustments to the stimulation time of stage I and stage II.

**Figure 4:**
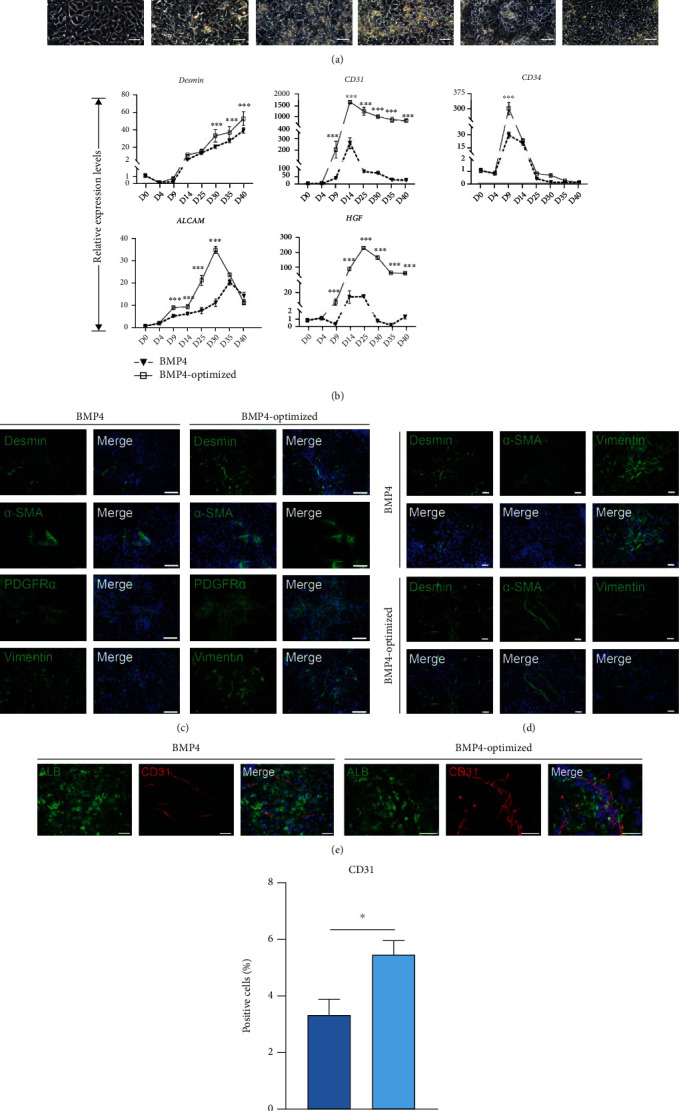
Optimization of the BMP4 differentiation protocol significantly promotes nonparenchymal cell production. (a) Schematic representation of the hepatocyte differentiation procedure during day 0 to day 30. The bright field images showed the sequential morphological changes (days 0-30) of hiPSC differentiation into hepatocytes. Scale bars, 50 *μ*m. (b) Quantitative PCR analysis showed the expression levels of endothelial lineage markers.  ^∗^*p* < 0.05,  ^∗∗^*p* < 0.01,  ^∗∗∗^*p* < 0.001, and *n* = 3. Immunofluorescence showed the differentiation of hiPSC-derived endothelial lineage in day 9 (c) and day14 (d). Scale bars, 50 *μ*m. (e, f) Immunofluorescence results showed expression CD31 in both differentiation protocols. Scale bars, 75 *μ*m.  ^∗^*p* < 0.05,  ^∗∗^*p* < 0.01,  ^∗∗∗^*p* < 0.001, and *n* = 3.

The optimized induction protocol and sequential morphological changes during differentiation day 0 to day 30 are shown in Figure 4(a) and Supplemental Figure 4A. The q-PCR analysis showed that the cells in BMP4 optimized induction medium have higher expression levels of mesoderm-related genes (*Desmin*, *CD31*, *CD34*, *ALCAM*, and *HGF*) than those in the original medium during hepatocyte differentiation (Figure 4(b), Supplemental Figure 4B). Immunofluorescence assay showed the cells expressing nonparenchymal marker at day 14 and day 25 (Figures 4(c) and 4(d), Supplemental Figure 4D). Immunofluorescence results showed expression of vascular maturation marker (CD31) in both differentiation protocols (Figures 4(e) and 4(f)).

### 3.5. Optimization of the BMP4 Differentiation Protocol Significantly Promotes Hepatocyte Function

**Figure 5:**
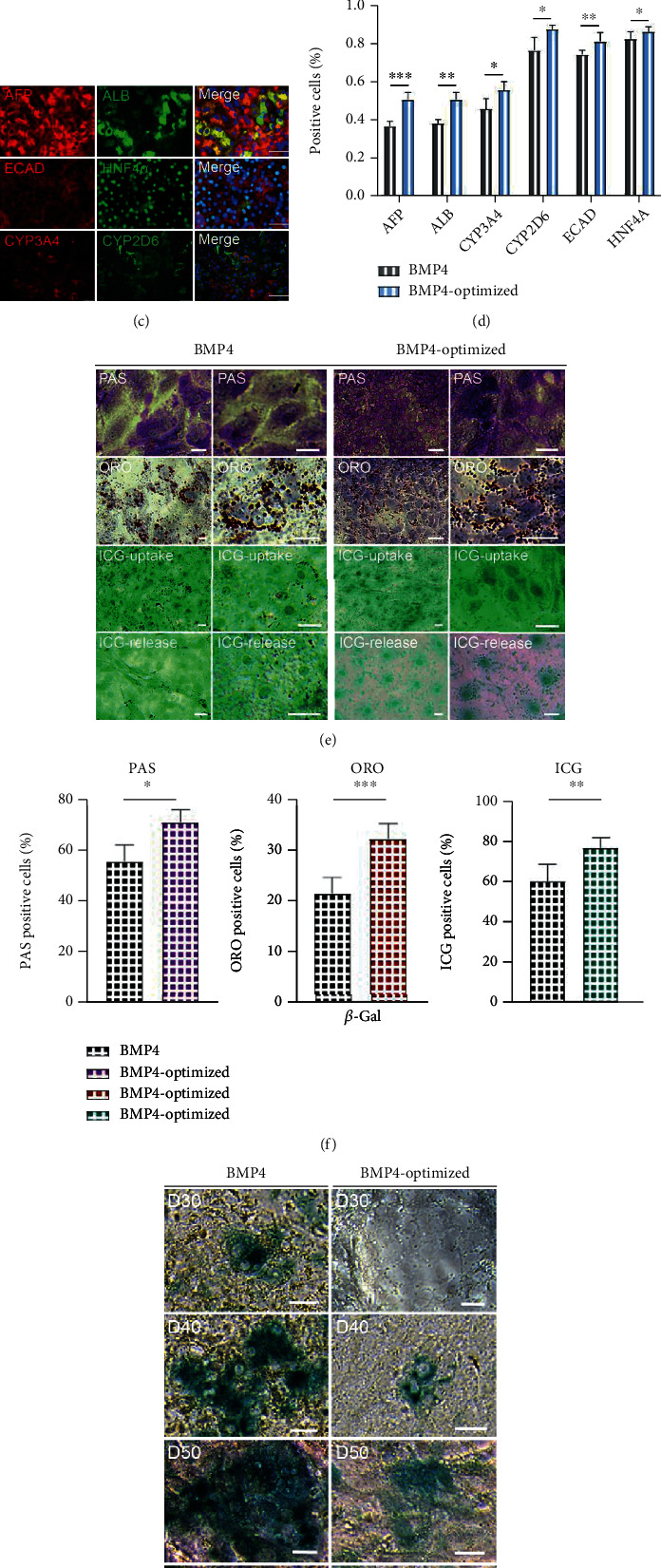
Optimization of the BMP4 differentiation protocol significantly promotes hepatocyte function. (a) q-PCR analysis showed the expression levels of mature hepatocyte markers.  ^∗^*p* < 0.05,  ^∗∗^*p* < 0.01,  ^∗∗∗^*p* < 0.001, and *n* = 3. (b–d) Immunofluorescence showed that the cells coexpressed hepatocyte markers induced by BMP4 or BMP4-optimized protocols. Scale bars, 50 *μ*m.  ^∗^*p* < 0.05,  ^∗∗^*p* < 0.01,  ^∗∗∗^*p* < 0.001, and *n* = 3. (e, f) Hepatocytes performed PAS (top), ORO (middle), and ICG (bottom). Scale bars, 50 *μ*m.  ^∗^*p* < 0.05,  ^∗∗^*p* < 0.01,  ^∗∗∗^*p* < 0.001, and *n* = 3. (g, h) Phase-contrast images of senescence *β*-Gal staining in hepatocytes during days 30-60 induced by original or BMP4-optimized medium. Scale bars, 25 *μ*m.  ^∗^*p* < 0.05,  ^∗∗^*p* < 0.01,  ^∗∗∗^*p* < 0.001, and *n* = 3. (i) ORO staining treated by BMP4 optimized medium in day 65. Scale bars, 50 *μ*m. (j) Immunofluorescence showed that hepatocytes coexpressed ALB and CYP3A4 in day 65. Scale bars, 25 *μ*m. (k) Production of ALB and AAT in day 35 hepatocytes. Primary hepatocytes were used as positive controls; hiPSCs were used as negative control.  ^∗^*p* < 0.05,  ^∗∗^*p* < 0.01,  ^∗∗∗^*p* < 0.001, and *n* = 3.

To confirm whether the hepatic cells in optimized induction protocol exhibited better liver function, we examined gene and protein levels during differentiation. The q-PCR results showed that the hepatic cells induced in the optimized medium expressed higher liver maturation genes (*ALB*, *AAT*, *HNF4α*, *CYP3A4*, and *AFP*) compared to the original medium (Figure 5(a), Supplemental Figure 4C). Immunofluorescence showed that hepatocytes both expressed hepatic maturation markers cultured in BMP4 or BMP4-optimized protocol at day 25 (Figures 5(b)–5(d)).

To further validate this result, we performed the analysis on hepatocyte function. In the BMP4-optimized group, there are more lipid droplets and glycogen synthesis ability than those in the BMP4 group. ICG staining results also showed the hepatocytes cultured in the BMP4-optimized medium showed higher ICG uptake and release capacity than the BMP4 group. This result indicated that the hepatocytes underwent BMP4-optimized treatment exhibited better hepatocyte function compared to the BMP4 group (Figures 5(e) and 5(f) and Supplemental Figure 4E). Phase-contrast staining images showed less *β*-Gal^+^ cells in the optimized group than those in the original BMP4 group for hepatic cells during differentiation (Figures 5(g) and 5(h)). These results were further confirmed by bright field images and TUNEL staining (Supplemental Figure 5). Hepatic cells induced in optimized medium still have the ability to metabolize lipids up to day 65 (Figure 5(i)). Immunofluorescence assay showed that hepatic cells at this time still expressed ALB and CYP3A4 (Figure 5(j)). To characterize the function of hepatocytes in different culture conditions, the ALB and AAT secretion ability of hepatocytes was detected by ELISA. The results showed that the ALB and AAT secretion capacity of hepatocytes in the BMP4 optimized group was significantly higher than that in the BMP4 group (Figure 5(k)).

w?>In conclusion, we optimized hepatocyte differentiation medium, which enables achieved mesoendodermal cell codifferentiation, and may also promote hepatocyte functional maturation and prolong survival time *in vitro*.

## 4. Discussion

The hepatocytes derived from hiPSCs have opened up new avenues in the field of regenerative medicine, including autologous cell therapies, and provide opportunities for drug development and studies of disease mechanisms [8]. One of the major drawbacks with hiPSC-derived hepatocytes is their decreased phenotypic maturity with lower liver-specific enzyme activities compared with human primary hepatocytes [24]. This limited maturity has restricted their utility in drug development and studies of disease mechanisms. To compensate for these deficiencies, many kinds of *in vitro* culture protocols for hepatocyte differentiation have been reported.

In 2013, Takebe et al. reported the first 3D liver bud organoids through mixed iPSC-derived hepatocytes, with human umbilical cord-derived umbilical vein endothelial cells and MSCs in a 5 :  4 : 1 ratio [25]. The 3D liver bud organoids self-assembled by three types of cells and connected to the host vascular system to form a functional vascularization after transplanted into host. Song et al. cocultured human adipose microvascular endothelial cells with hiPSC in a 1 : 3 ratio and eventually obtained hepatocytes with higher specific gene expression and better liver function *in vitro*, which were transplanted into mice for prolonged albumin secretion [17]. These reports demonstrated the important role of intercellular interactions and signaling for liver differentiation, while highlighting the importance of the physiological microenvironment for mimicking the liver during embryonic development. Notably, although multicellular codifferentiation has a promising contribution to hepatocytes, the introduction of exogenous cells during this process may lead to immune rejection. In addition, certain cell types used for multicellular codifferentiation are more difficult to culture, such as hepatic stellate cells. There is another recent study by engineering a wide range of Gata6 expression levels in a pluripotent cell population, through directing their differentiation into a heterogeneous tissue and detecting a liver bud-like structure containing stromal cells, vascular tube-like structures and haematopoiesis-like processes [26, 27]. The tissue generated by overexpressed Gata6 is embryonic in nature, and while further maturation *in* or *ex vivo* will be necessary to achieve the full functionality of an adult organ [26]. However, these studies artificially combined cells that were isolated from multiple individuals and genetically engineered pulse of protein expression, suggesting a severe limitation of the transplanting applications based on these strategies.

To achieve hepatocytes with long-term or permanent function and engraftment, a much improved protocol will be required that can either completely escape the foreign body reaction or become functionally vascularized. Recently, based on the published monolayer hepatocyte differentiation protocol [16], we investigated the effects of two hepatocyte culture media (HZM and HCM) on the hepatocyte maturation stage. The results demonstrated that HCM medium enhances the maturation and functional properties of hiPSC-derived hepatocytes. As we all know, BMP4 is required for the generation of a multipotent mesoderm progenitor population from human ESCs [28]. Therefore, BMP4 is widely used in the differentiation of hepatic nonparenchymal cells [19, 20, 29]. In order to mimic the embryonic development of the liver, we used BMP4 in the early stage of the differentiation protocol. This leads to codifferentiation of endodermal-derived hepatocytes with mesodermal-derived nonparenchymal cells, such as hepatic stellate cells and vascular endothelial cells. Unfortunately, the hepatocytes obtained by this method are too matured to have a longer life span. Subsequently, we further optimized the codifferentiation protocol and adjusted the duration of stimulation for each stage. As a result, hepatocytes had significantly improved their maturity and prolonged their lifespan, which could maintain liver function for at least 65 days.

In conclusion, we have successfully developed the codifferentiation system with endoderm-derived hepatocytes and mesodermal-nonparenchymal cells, which may enhance the maturation of hepatocytes. The coordinated action and the mutual secretion of signals may enhance the maturity and functional properties of differentiated hepatocytes. However, the mechanism of hepatocyte function enhancement is still unknown and requires further research.

## Data Availability

The data used to support the findings of this study are included within the article.

## Supplementary Materials

Supplementary 1 Supplemental Figure 1: we analyzed the pluripotency of hiPSCs (hiPSC-UC and hiPSC-WD) and found that almost all cells stained positive for alkaline phosphatase (Supplemental Figure 1A). We also examined the pluripotency in two types of hiPSCs at day 0 by flow cytometry and found that more than 90% cells coexpressed SSEA4 and TRA-1-81 (Supplemental Figure 1B, C). This result was confirmed by immunofluorescence (Supplemental Figure 1D). The statistics of ORO, PAS, and ICG showed that the function of hepatocytes cultured in HCM medium is stronger than that of HZM medium (Supplemental Figure 1E). And the observed data showed that the HCM group significantly slowed down the senescence of hepatocytes (Supplemental Figure 1F). Supplemental Figure 2. to validate our findings, we performed an additional experiment using hiPSC-WD in this study. Schematic of hepatocyte induction and sequential morphological changes is outlined in Supplement Figure 2A. The gene expression levels of hepatocyte markers (*AFP*, *ALB*, *CK18*, *AAT*, *HNF4α*, and *CYP3A4*) in hiPSC-WD derived-hepatocytes were significantly increased by using HCM compared to HZM (Supplemental Figure 2B). Immunofluorescence results showed coexpression of sevaral proteins (AFP, ALB, and CYP3A4) in day 25 (Supplemental Figure 2C). Sebsquently, we also used hiPSC-WD to anlyzed the hepatocyte function cultured in HCM or HZM media via periodic acid-Schiff (PAS), Oil Red O (ORO), and indocyanine green (ICG) staining in day 25 (Supplemental Figure 2D). ELISA showed that the amount of secreted ALB and AAT was more strongly in the HCM group (Supplemental Figure 2E). These results also indicated that HCM may be more suitable for maintenance of hiPSC-derived hepatocytes. Supplemental Figure 3: our BMP4 induction protocol to generate hiPSC-WD-derived hepatocytes and sequential morphological changes is outlined in Supplemental Figure 3A. We also detected whether mesodermal-derived cells are accompanied with hepatocyte differentiation through q-RCR and immunofluorescence analysis. The q-RCR result showed that the expression of endothelial cell markers (*ALCAM*, *Desmin*, *HGF*, *CD34*, and *CD31*) and hepatocyte makers (*AFP*, *ALB*, *AAT*, *HNF4α*, and *CK18*) gradually increases, and their expression levels were higher in the Wnt3a-treated group during hepatocyte differentiation (Supplemental Figure 3B, C). Immunofluorescence showed that endothelial cells (Desmin-positive cells) were present in both Wnt3a- and BMP4-induced conditions in hepatocyte stage (Supplemental Figure 3D). To compare the live function of the two-differentiation protocol, ICG, PAS, and ORO staining were performed on the hepatocytes in day 30. Our results showed that ICG, PAS, and ORO staining in the BMP4 group were significantly stronger than that in the Wnt3a group (Supplement Figure 3E). Supplemental Figure 4: our BMP4-optimized induction protocol to generate hiPSC-WD-derived hepatocytes and sequential morphological changes is outlined in Supplemental Figure 4A. We also detected whether mesodermal-derived cells are accompanied with hepatocyte differentiation through q-RCR and immunofluorescence analysis. The q-RCR result showed that the expression of endothelial cell markers (*ALCAM*, *Desmin*, *HGF*, *CD34*, and *CD31*) and hepatocyte makers (*AFP*, *ALB*, *AAT*, *HNF4α*, and *CK18*) gradually increases, and their expression levels were higher in the BMP4-optimized group during hepatocyte differentiation (Supplemental Figure 4B, C). Immunofluorescence showed that endothelial cells (Desmin-positive cells) were present in both BMP4 and BMP4-optimized induced conditions in hepatocyte stage (Supplemental Figure 4D). To compare the live function of the two-differentiation protocol, ICG, PAS, and ORO staining were performed on the hepatocytes in day 30. Our results showed that ICG, PAS, and ORO staining in the BMP4-optimized group were significantly stronger than that in the BMP4 group. Supplemental Figure 5: bright field images showed that the hepatocytes induced by BMP4 protocol gradually lose their polygonal morphology during days 40-60 (Supplemental Figure 5A). Representative TUNEL staining images show fewer stained cells in the BMP4-optimized relative to the BMP4 group at day 50 (Supplemental Figure 5B).

Supplementary 2 Supplemental Materials. The complete list of the primary and secondary antibodies used is provided in supplemental materials (Table S1). Primers used are listed in supplemental materials (Table S2).
