# Supplementary material for: Generation of Hepatocytes and Nonparenchymal Cell Codifferentiation System from Human-Induced Pluripotent Stem Cells
Source: Stem Cells Int. 2022 Nov 22;2022:3222427. doi: 10.1155/2022/3222427 (PMC9709383; doi:10.1155/2022/3222427)
Supplement: Supplementary 1 — Supplemental Figure 1: we analyzed the pluripotency of hiPSCs (hiPSC-UC and hiPSC-WD) and found that almost all cells stained positive for alkaline phosphatase (Supplemental Figure 1A). We also examined the pluripotency in two types of hiPSCs at day 0 by flow cytometry and found that more than 90% cells coexpressed SSEA4 and TRA-1-81 (Supplemental Figure 1B, C). This result was confirmed by immunofluorescence (Supplemental Figure 1D). The statistics of ORO, PAS, and ICG showed that the function of hepatocytes cultured in HCM medium is stronger than that of HZM medium (Supplemental Figure 1E). And the observed data showed that the HCM group significantly slowed down the senescence of hepatocytes (Supplemental Figure 1F). Supplemental Figure 2. to validate our findings, we performed an additional experiment using hiPSC-WD in this study. Schematic of hepatocyte induction and sequential morphological changes is outlined in Supplement Figure 2A. The gene expression levels of hepatocyte markers (AFP, ALB, CK18, AAT, HNF4α, and CYP3A4) in hiPSC-WD derived-hepatocytes were significantly increased by using HCM compared to HZM (Supplemental Figure 2B). Immunofluorescence results showed coexpression of sevaral proteins (AFP, ALB, and CYP3A4) in day 25 (Supplemental Figure 2C). Sebsquently, we also used hiPSC-WD to anlyzed the hepatocyte function cultured in HCM or HZM media via periodic acid-Schiff (PAS), Oil Red O (ORO), and indocyanine green (ICG) staining in day 25 (Supplemental Figure 2D). ELISA showed that the amount of secreted ALB and AAT was more strongly in the HCM group (Supplemental Figure 2E). These results also indicated that HCM may be more suitable for maintenance of hiPSC-derived hepatocytes. Supplemental Figure 3: our BMP4 induction protocol to generate hiPSC-WD-derived hepatocytes and sequential morphological changes is outlined in Supplemental Figure 3A. We also detected whether mesodermal-derived cells are accompanied with hepatocyte differentiation through [file 3222427.f1.docx]

**Generation of hepatocytes and non-parenchymal cells co-differentiation system from human induced pluripotent stem cells**

Ying Shi^1^, Jiali Deng^1^, Xiaopu Sang^2^, Yihang Wang^1^, Fei He^3^, Xiaoni Chen^3^, Anlong Xu^1, 2, *^, Fenfang Wu^1, 3, *^

^1^State Key Laboratory of Biocontrol, Guangdong Province Key Laboratory of Pharmaceutical Functional Genes, College of Life Sciences, Sun Yat-Sen University, Guangzhou, China; ^2^School of Life Sciences, Beijing University of Chinese Medicine, Beijing, China; ^3^Department of Central Laboratory, Shenzhen Hospital, Beijing University of Chinese Medicine, Shenzhen, China.

*Correspondence: Fenfang Wu ([wufenfang19@126.com](mailto:wufenfang19@126.com)); Anlong Xu ([xuanlong@bucm.edu.cn](mailto:xuanlong@bucm.edu.cn))

We analysis the pluripotency of hiPSCs (hiPSC-UC and hiPSC-WD), and found that almost all cells stained positive for alkaline phosphatase **(Supplemental Fig. 1A**). We also examined the pluripotency in two types of hiPSCs at day 0 by ﬂow cytometry and found that more than 90% cells co-expressed SSEA4 and TRA-1-81 (**Supplemental Fig. 1B, C**). This result was conﬁrmed by immunoﬂuorescence (**Supplemental Fig. 1D**).

The statistics of ORO, PAS and ICG showed that the function of hepatocytes cultured in HCM medium is stronger than that of HZM medium (**Supplemental Fig. 1E**). And the observed data showed that HCM group signiﬁcantly slowed down the senescence of hepatocytes (**Supplemental Fig. 1F**).


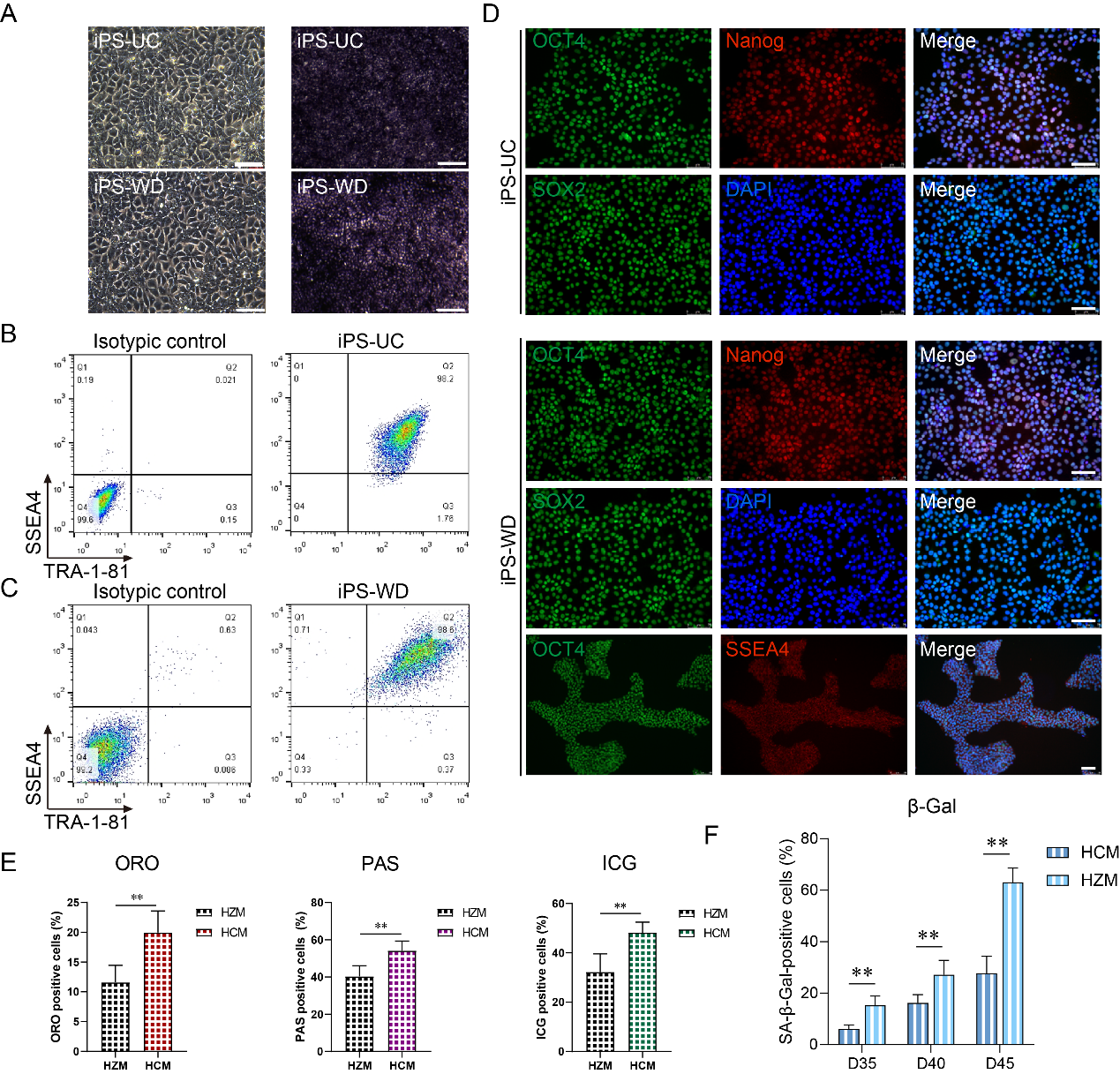


**Supplemental Figure 1. Analysis the pluripotency of iPSCs-UC and iPSCs-WD.** (A) Brightfield image and alkaline phosphatase staining of iPSC-UC and iPSC-WD. Scale bar: 100 μm. (B, C) SSEA4 and TRA-1-81-positive cells detected by flow cytometry in iPSC-UC and iPSC-WD. (D) HiPSCs (hiPSC-UC and hiPSC-WD) expression of NANOG, OCT4, SOX2 and SSEA4 confirmed by immunofluorescence. Scale bar: 75 μm. (E) Statistics of ORO, PAS, and ICG in each group. **p* <0.05; ***p* <0.01; ****p* <0.001; n=3. (F) Quantiﬁcation of β-Gal staining showed that HCM group signiﬁcantly slowed down hepatocyte senescence.

To validate our findings, we performed an additional experiment using hiPSC-WD in this study. Schematic of hepatocyte induction and sequential morphological changes is outlined in **Supplement Fig. 2A**. The gene expression levels of hepatocyte markers (*AFP*, *ALB*, *CK18*, *AAT*, *HNF4α*, and *CYP3A4*) in hiPSC-WD derived-hepatocytes were significantly increased by using HCM compared to HZM **(Supplemental Fig. 2B)**. Immunofluorescence results showed co-expression of sevaral proteins (AFP, ALB and CYP3A4) in day 25 **(Supplemental Fig. 2C)**. Sebsquently, we also used hiPSC-WD to anlyzed the hepatocytes function cultured in HCM or HZM media via periodic acid-schiff (PAS), Oil red O (ORO) and indocyanine green (ICG) staining in day 25 **(Supplemental Fig. 2D)**. ELISA assay showed that the amount of secreted ALB and AAT was more strongly in HCM group **(Supplemental Fig. 2E)**. These results also indicated that HCM may be more suitable for maintenance of hiPSCs derived-hepatocytes.


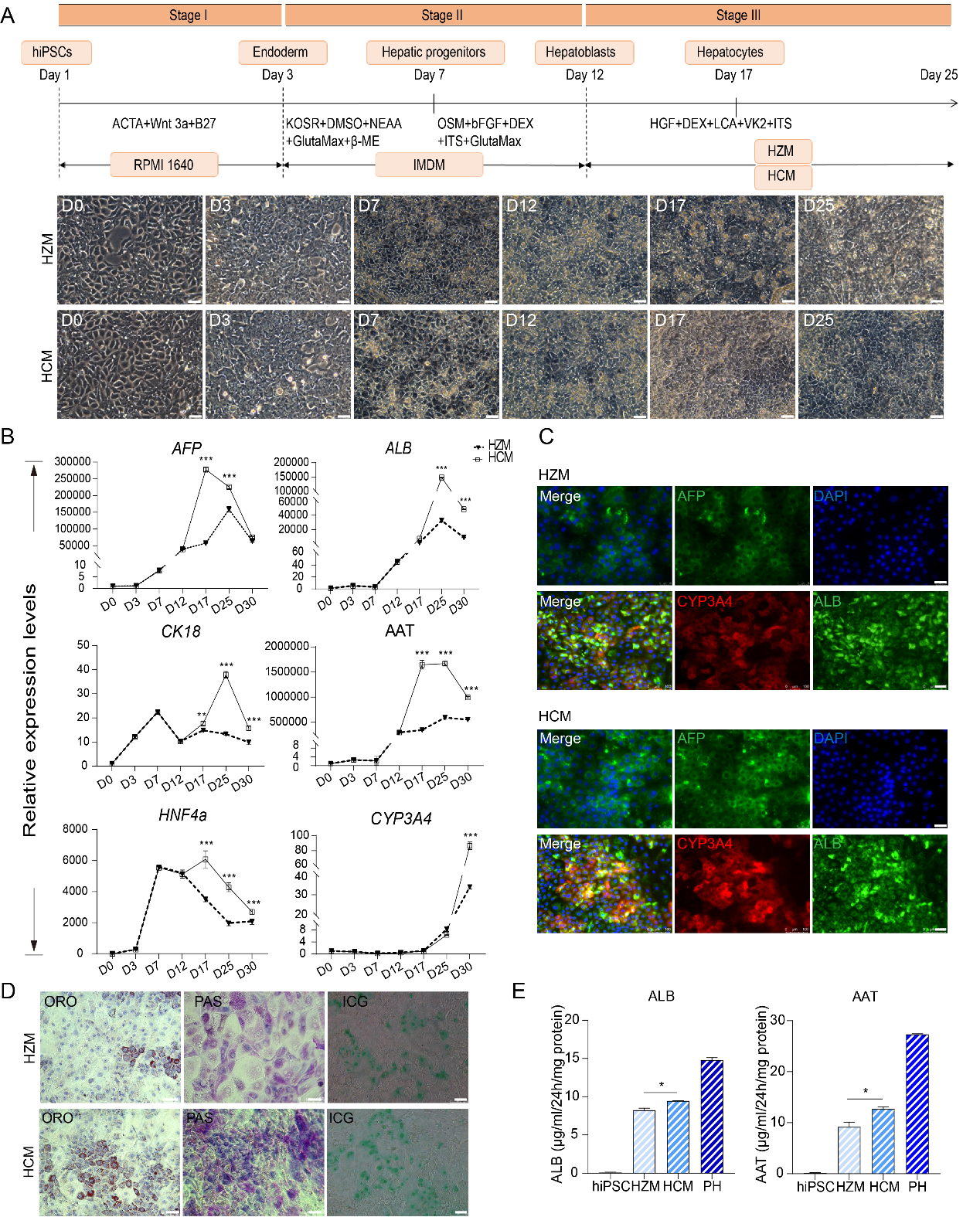


**Supplemental Figure 2. Screening of an optimal culture medium for maintenance of hiPSC-WD derived hepatocytes.** (A) Schematic representation of the hepatocytes differentiation procedure during day 0 to day 30. The bright field images showed the sequential morphological changes (day 0-30) of hiPSC-WD differentiation into hepatocytes. Scale bars, 50μm. (B) Q-PCR analysis showed that the expression levels of mature hepatocytes markers. **p* <0.05; ***p* <0.01; ****p* <0.001; n=3. (C) Immunofluorescence showed that the cells co-expressed hepatocytes markers induced by HZM or HCM optimized protocols. (D) Statistics of ORO, PAS, ICG and β-Gal in each group. (E) Production of ALB and AAT in day-35 hepatocytes. Primary hepatocytes were used as positive controls; hiPSCs were used as negative control. **p* <0.05; ***p* <0.01; ****p* <0.001; n=3.

Our BMP4 induction protocol to generate hiPSC-WD derived hepatocytes, and sequential morphological changes is outlined in **Supplemental Fig. 3A**. We also detected whether mesodermal-derived cells are accompanied with hepatocytes differentiation through q-RCR and immunoﬂuorescence analysis. The q-RCR result showed that the expression of endothelial cell markers (*ALCAM*, *Desmin*, *HGF*, *CD34* and *CD31*) and hepatocyte maker (*AFP*, *ALB*, *AAT*, *HNF4α* and *CK18*) gradually increases, and their expression levels were higher in the Wnt3a-treated group during hepatocytes differentiation (**Supplemental Fig 3B, C**). Immunoﬂuorescence showed that endothelial cells (Desmin positive cells) were present in both Wnt3a and BMP4 induced conditions in hepatocyte stage (**Supplemental Fig 3D**).

To compare the live function of the two differentiation protocol, ICG, PAS and ORO staining were performed on the hepatocytes in day 30. Our results showed that ICG, PAS and ORO staining in BMP4 group were significantly stronger than Wnt3a group (**Supplement Fig 3E**).


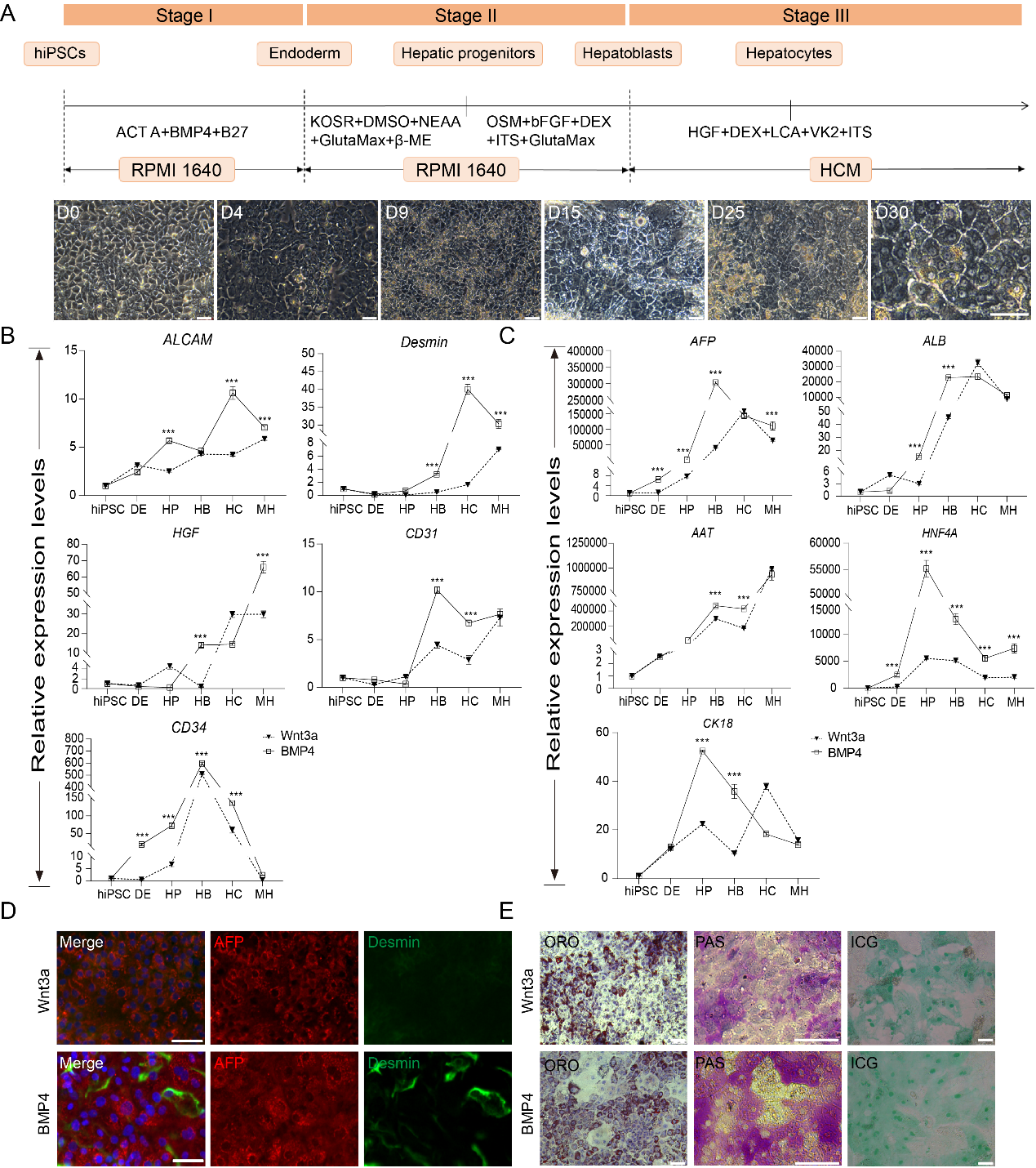


**Supplemental Figure 3.** **Hepatocytes functional assay after hepatocytes and non-parenchymal cells co-differentiation in hiPSC-WD cells.** (A) Schematic representation of the hepatocytes differentiation procedure during day 0 to day 30. The bright field images showed the sequential morphological changes (day 0-30) of hiPSCs differentiation into hepatocytes. Scale bars, 50μm. (B, C) Q-PCR analysis showed that the expression levels of mature hepatocytes markers. * *P* < 0.05, ** *P* < 0.01, *** *P* < 0.001, n=3. (D) Immunofluorescence showed that the cells co-expressed hepatocytes markers induced by Wnt3a or BMP4 protocol. Scale bar, 50μm. (E) Staining of ORO, PAS, ICG and β-Gal in each group.

Our BMP4 optimized induction protocol to generate hiPSC-WD derived hepatocytes, and sequential morphological changes is outlined in **Supplemental Fig 4A**. We also detected whether mesodermal-derived cells are accompanied with hepatocytes differentiation through q-RCR and immunoﬂuorescence analysis. The q-RCR result showed that the expression of endothelial cell markers (*ALCAM*, *Desmin*, *HGF*, *CD34* and *CD31*) and hepatocyte maker (*AFP*, *ALB*, *AAT*, *HNF4α* and *CK18*) gradually increases, and their expression levels were higher in the BMP4-optimized group during hepatocytes differentiation (**Supplemental Fig 4B, C**). Immunoﬂuorescence showed that endothelial cells (Desmin positive cells) were present in both BMP4 and BMP4-optimized induced conditions in hepatocyte stage (**Supplemental Fig 4D**).

To compare the live function of the two differentiation protocol, ICG, PAS and ORO staining were performed on the hepatocytes in day 30. Our results showed that ICG, PAS and ORO staining in BMP4-optimized group were significantly stronger than BMP4 group.

_
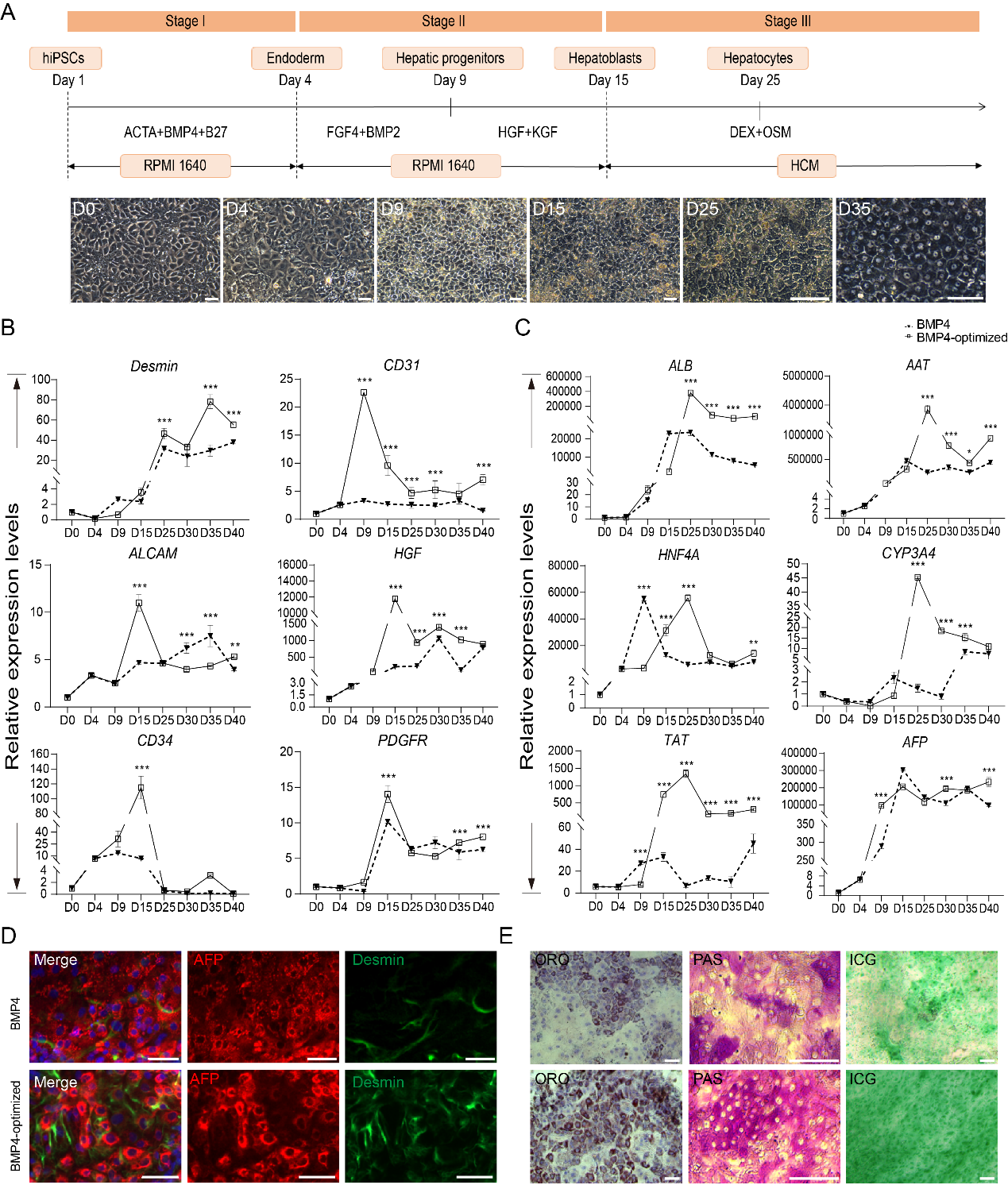
_

**Supplemental Figure 4. Optimization of the BMP4 differentiation protocol significantly promotes hepatocyte function in hiPSC-WD cells.** (A) Schematic representation of the hepatocytes differentiation procedure during day 0 to day 30. The bright field images showed the sequential morphological changes (day 0-30) of hiPSC-WD differentiation into hepatocytes. Scale bars, 50μm. (B, C) Q-PCR analysis showed that the expression levels of mature hepatocytes markers. **p* <0.05; ***p* <0.01; ****p* <0.001; n=3. (D) Immunofluorescence showed that the cells co-expressed hepatocytes markers induced by BMP4 or BMP4 optimized protocols. Scale bars, 50μm. (E) Staining of ORO, PAS, and ICG in each group.

Bright field images showed that the hepatocytes induced by BMP4 protocol gradually lose their polygonal morphology during day 40-60 (**Supplemental Fig 5A**). Representative TUNEL staining images show fewer stained cells in the BMP4 optimized relative to the BMP4 group at day 50 (**Supplemental Fig 5B**).

_
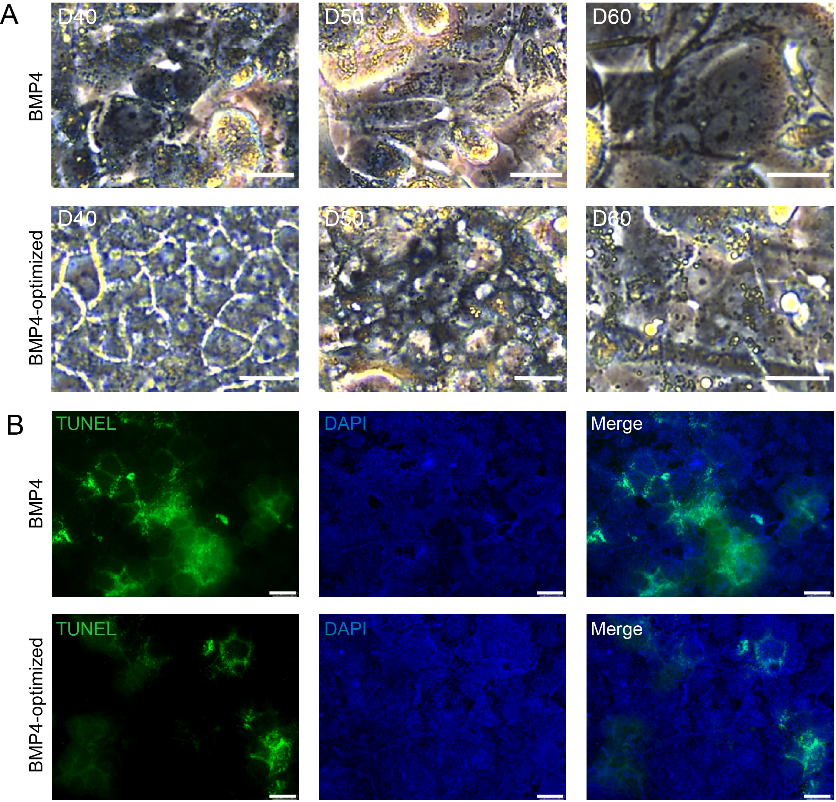
_

**Supplemental Figure 5.** (A) Bright field images of senescence in hepatic cells during day 40-60 treated with BMP4 or BMP4-optimized. Scale bars, 25μm. (B) Representative TUNEL and DAPI staining images are shown at day 50. Scale bars, 500μm.

Table S1 Immunofluorescence antibodies

| Name | Supplier | Cat no. | Host | dilution ratio |
| --- | --- | --- | --- | --- |
| Anti-NANOG antibody | Abcam | Ab109250 | Mouse | 1:200 |
| Anti-OCT4 antibody | Abcam | Ab184665 | Rabbit | 1:200 |
| Anti-SSEA4 antibody | GeneTex | GTX48037 | Mouse | 1:200 |
| Anti-SOX17 antibody | Abcam | Ab84990 | Mouse | 1:100 |
| Anti-FOXA2 antibody | Abcam | Ab214449 | Rabbit | 1:200 |
| Anti-T antibody | Abcam | Ab209665 | Rabbit | 1:200 |
| Anti-Desmin antibody | Abcam | ab32362 | Rabbit | 1:250 |
| Anti-α-SMA antibody | CST | 19245S | Rabbit | 1:250 |
| Anti-PDGFRα antibody | CST | 3174S | Rabbit | 1:250 |
| Anti-Vimentin antibody | CST | 5741 | Rabbit | 1:250 |
| Anti-AFP antibody | GeneTex | GTX15650 | Mouse | 1:200 |
| Anti-ALB antibody | Abcam | Ab207327 | Rabbit | 1:200 |
| Anti-CYP3A4 antibody | GeneTex | GTX60577 | Mouse | 1:200 |
| Anti-CYP2D6 antibody | Abcam | Ab185625 | Rabbit | 1:200 |
| Anti-CK18 antibody | Abcam | Ab133263 | Rabbit | 1:200 |
| Anti-E-CAD antibody | Abcam | Ab1416 | Mouse | 1:100 |
| Anti-HNF4α antibody | Abcam | Ab92378 | Rabbit | 1:100 |
| Donkey anti-Rabbit IgG Alexa Fluor 488 | Invitrogen | A21206 | Donkey | 1:500 |
| Donkey anti-Mouse IgG Alexa Fluor 594 | Invitrogen | A21203 | Donkey | 1:500 |

Table S2 Primer sequence for qPCR

| Name | F/R | Primer sequence |
| --- | --- | --- |
| *CD31* | F  R | AGGCCCCAATACACTTCACA  CGGGGAATTCCAGTATCAC |
| *CD34* | F  R | GCCATTCAGCAAGACAACAC  AAGGGTTGGGCGTAAGAGAT |
| *Desmin* | F  R | GAAGCTGCTGGAGGGAGAG  ATGGACCTCAGAACCCCTTT |
| *HGF* | F  R | CGCTGGGAGTACTGTGCAAT  CCCTGTAGCCTTCTCCTTGA |
| *ALCAM* | F  R | CTTCTGCCTCTTGATCTCCG  AGGTACGTCAAGTCGGCAAG |
| *EMOES* | F  R | TTAGTGGGTGGATGGGGATAAAG  CCAAAAACTACTCCCCTAACTACATAC |
| *T* | F  R | AGCCAAAGACAATCAGCAGAAA  CACAAAAGGAGGGGCTTCACTA |
| *MIXL1* | F  R | TGCTTTCAAAACACTCGAGGAC  GAGTGATCGAAGTAACAGGTGC |
| *EVX1* | F  R | CAAATCCTCACTCC ACACTC A  GAAGAACCACTCCCTCTCAGT C |
| *HNF4α* | F  R | TGTACTCCTGCAGATTTAGCC  CTGTCCTCATAGCTTGACCT |
| *AFP* | F  R | AGTGAGGACAAACTATTGGCCT  ACACCAGGGTTTACTGGAGTC |
| *ALB* | F  R | GAGACCAGAGGTTGATGTGATG  AGTTCCGGGGCATAAAAGTAAG |
| *AAT* | F  R | CTTCTCCCCAGTGAGCATCG  TGGATCTGAGCCTCCGGAAT |
| *CYP3A4* | F  R | GGTGGTGAATGAAACGCTCAG  CACCCCTTTGGGAATGAACA |
| *CK18* | F  R | TCGCAAATACTGTGGACAATGC  GCAGTCGTGTGATATTGGTGT |
