# Supplementary material for: Generation of Hepatocytes and Nonparenchymal Cell Codifferentiation System from Human-Induced Pluripotent Stem Cells
Source: Stem Cells Int. 2022 Nov 22;2022:3222427. doi: 10.1155/2022/3222427 (PMC9709383; doi:10.1155/2022/3222427)
Supplement: Supplementary 2 — Supplemental Materials. The complete list of the primary and secondary antibodies used is provided in supplemental materials (Table S1). Primers used are listed in supplemental materials (Table S2). [file 3222427.f2.docx]

Table S1 Immunofluorescence antibodies

| Name | Supplier | Cat no. | Host | dilution ratio |
| --- | --- | --- | --- | --- |
| Anti-NANOG antibody | Abcam | Ab109250 | Mouse | 1:200 |
| Anti-OCT4 antibody | Abcam | Ab184665 | Rabbit | 1:200 |
| Anti-SSEA4 antibody | GeneTex | GTX48037 | Mouse | 1:200 |
| Anti-SOX17 antibody | Abcam | Ab84990 | Mouse | 1:100 |
| Anti-FOXA2 antibody | Abcam | Ab214449 | Rabbit | 1:200 |
| Anti-T antibody | Abcam | Ab209665 | Rabbit | 1:200 |
| Anti-Desmin antibody | Abcam | ab32362 | Rabbit | 1:250 |
| Anti-α-SMA antibody | CST | 19245S | Rabbit | 1:250 |
| Anti-PDGFRα antibody | CST | 3174S | Rabbit | 1:250 |
| Anti-Vimentin antibody | CST | 5741 | Rabbit | 1:250 |
| Anti-AFP antibody | GeneTex | GTX15650 | Mouse | 1:200 |
| Anti-ALB antibody | Abcam | Ab207327 | Rabbit | 1:200 |
| Anti-CYP3A4 antibody | GeneTex | GTX60577 | Mouse | 1:200 |
| Anti-CYP2D6 antibody | Abcam | Ab185625 | Rabbit | 1:200 |
| Anti-CK18 antibody | Abcam | Ab133263 | Rabbit | 1:200 |
| Anti-E-CAD antibody | Abcam | Ab1416 | Mouse | 1:100 |
| Anti-HNF4α antibody | Abcam | Ab92378 | Rabbit | 1:100 |
| Donkey anti-Rabbit IgG Alexa Fluor 488 | Invitrogen | A21206 | Donkey | 1:500 |
| Donkey anti-Mouse IgG Alexa Fluor 594 | Invitrogen | A21203 | Donkey | 1:500 |

Table S2 Primer sequence for qPCR

| Name | F/R | Primer sequence |
| --- | --- | --- |
| *CD31* | F  R | AGGCCCCAATACACTTCACA  CGGGGAATTCCAGTATCAC |
| *CD34* | F  R | GCCATTCAGCAAGACAACAC  AAGGGTTGGGCGTAAGAGAT |
| *Desmin* | F  R | GAAGCTGCTGGAGGGAGAG  ATGGACCTCAGAACCCCTTT |
| *HGF* | F  R | CGCTGGGAGTACTGTGCAAT  CCCTGTAGCCTTCTCCTTGA |
| *ALCAM* | F  R | CTTCTGCCTCTTGATCTCCG  AGGTACGTCAAGTCGGCAAG |
| *EMOES* | F  R | TTAGTGGGTGGATGGGGATAAAG  CCAAAAACTACTCCCCTAACTACATAC |
| *T* | F  R | AGCCAAAGACAATCAGCAGAAA  CACAAAAGGAGGGGCTTCACTA |
| *MIXL1* | F  R | TGCTTTCAAAACACTCGAGGAC  GAGTGATCGAAGTAACAGGTGC |
| *EVX1* | F  R | CAAATCCTCACTCC ACACTC A  GAAGAACCACTCCCTCTCAGT C |
| *HNF4α* | F  R | TGTACTCCTGCAGATTTAGCC  CTGTCCTCATAGCTTGACCT |
| *AFP* | F  R | AGTGAGGACAAACTATTGGCCT  ACACCAGGGTTTACTGGAGTC |
| *ALB* | F  R | GAGACCAGAGGTTGATGTGATG  AGTTCCGGGGCATAAAAGTAAG |
| *AAT* | F  R | CTTCTCCCCAGTGAGCATCG  TGGATCTGAGCCTCCGGAAT |
| *CYP3A4* | F  R | GGTGGTGAATGAAACGCTCAG  CACCCCTTTGGGAATGAACA |
| *CK18* | F  R | TCGCAAATACTGTGGACAATGC  GCAGTCGTGTGATATTGGTGT |
